# Mobility-Aware Service Caching in Mobile Edge Computing for Internet of Things

**DOI:** 10.3390/s20030610

**Published:** 2020-01-22

**Authors:** Hua Wei, Hong Luo, Yan Sun

**Affiliations:** Beijing Key Laboratory of Intelligent Telecommunications Software and Multimedia, Beijing University of Posts and Telecommunications, Beijing 100876, China; weihua2015@bupt.edu.cn (H.W.); sunyan@bupt.edu.cn (Y.S.)

**Keywords:** sensor networks, internet of things, mobility sensor, mobile edge computing, service cache, mobility-aware, service response time

## Abstract

The mobile edge computing architecture successfully solves the problem of high latency in cloud computing. However, current research focuses on computation offloading and lacks research on service caching issues. To solve the service caching problem, especially for scenarios with high mobility in the Sensor Networks environment, we study the mobility-aware service caching mechanism. Our goal is to maximize the number of users who are served by the local edge-cloud, and we need to make predictions about the user’s target location to avoid invalid service requests. First, we propose an idealized geometric model to predict the target area of a user’s movement. Since it is difficult to obtain all the data needed by the model in practical applications, we use frequent patterns to mine local moving track information. Then, by using the results of the trajectory data mining and the proposed geometric model, we make predictions about the user’s target location. Based on the prediction result and existing service cache, the service request is forwarded to the appropriate base station through the service allocation algorithm. Finally, to be able to train and predict the most popular services online, we propose a service cache selection algorithm based on back-propagation (BP) neural network. The simulation experiments show that our service cache algorithm reduces the service response time by about 13.21% on average compared to other algorithms, and increases the local service proportion by about 15.19% on average compared to the algorithm without mobility prediction.

## 1. Introduction

With the rapid development of large-scale cloud computing, more and more service providers have chosen to deploy content and services on the cloud. However, the traditional cloud computing environment is not suitable for Internet of Things (IoT) services [1], especially for delay-sensitive services that involve sensors [2], such as cognitive assistance, image recognition, mobile games and augmented/virtual reality (AR/VR) [3]. Generally speaking, such services are not only highly delay-sensitive, but also have complex computing requirements. For this problem, the researchers propose a mobile edge computing model, i.e., deploying an edge-cloud server on the base station which is close to the end user to perform some computing jobs. It can significantly reduce network transmission delay [4].

Based on the mobile edge computing (MEC) architecture, there are many related research [5], including computation offloading, energy efficiency, security mechanisms [6], etc. [7,8]. Now, the current research is mainly focus on computation offloading. The computation offloading means that the mobile device offloads its computing jobs to the edge-cloud network. It solves the problems of resource storage, computational performance and efficiency. However, service caching, another topic which is as important as computation offloading receives less attention.

In contrast to computation offloading, service caching refers to caching the corresponding service and its associated database/library in an edge-cloud server (for example, a MEC-enabled base station (BS)) so that it can perform the corresponding computing job [3]. It needs to download associated database/library/program from the remote cloud in advance. Service caching is a relatively long-term decision compared to computation offloading [9]. At the same time, since the edge-cloud server deployed on the BS is smaller than the remote cloud server, the edge-cloud resources and computing power are limited compared with the remote cloud center, so that any edge-cloud server cannot support a wide range of applications. Therefore, to make the local edge-cloud serve more users, we must decide which services should be cached.

To guarantee the quality of service, service providers (SP) often establish service level agreements (SLA) with customers to ensure quality of service. Among them, service response time, which defines the time from request generation to service result return, is the most important indicator in SLA. Therefore, an important problem in cloud computing is how to complete service deployment between multiple data centers to reduce the service response time. However, for delay-sensitive sensor services, the transmission time of the link is difficult to guarantee due to the long distance between the cloud centers. MEC architecture effectively solves this problem by deploying edge-cloud servers on base stations (BSs) close to the end user. To enable more users to get lower service response time, we should choose popular services for caching. Therefore, how to effectively determine popular services is one of the key problems in this paper.

At present, a small amount of research on service caching has focused on: joint optimization of computing offloading and how to coordinate service caching in dense cellular networks. There is a lack of consideration for user or sensor mobility. However, with the development of the 4/5G network, the coverage of a single BS is getting smaller and smaller. Due to the influence of terrain, the coverage radius of the BS ranges from 500 m to 1 km. In such a dense range, frequent switching of the BS will result in frequent switching of available edge-cloud servers, thereby affecting service performance. Therefore, we must consider the user mobility. In this paper, we propose a mobile-aware service caching mechanism in mobile edge computing.

**Figure 1:**
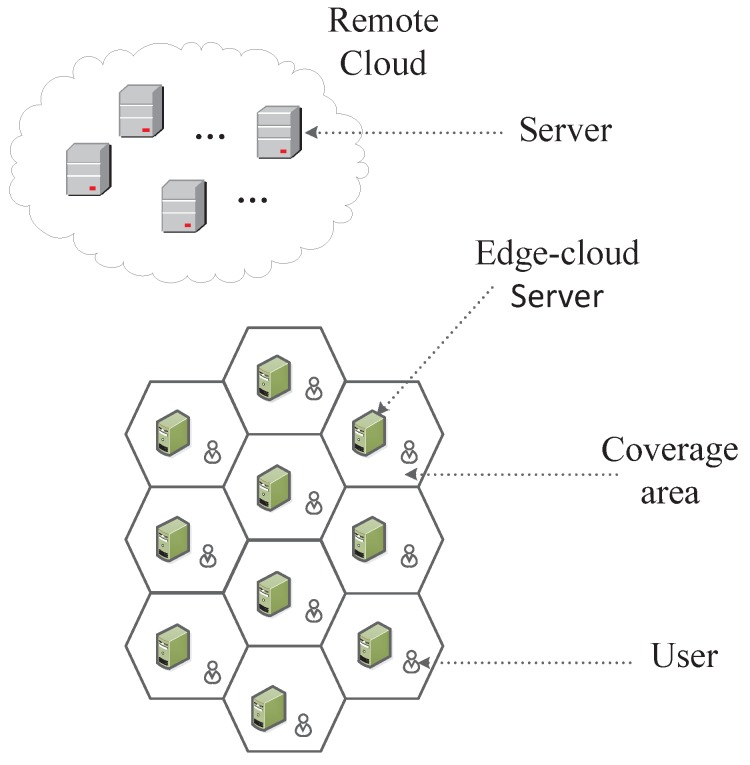
Traditional Edge-Cloud Computing Coverage Model.

**Figure 2:**
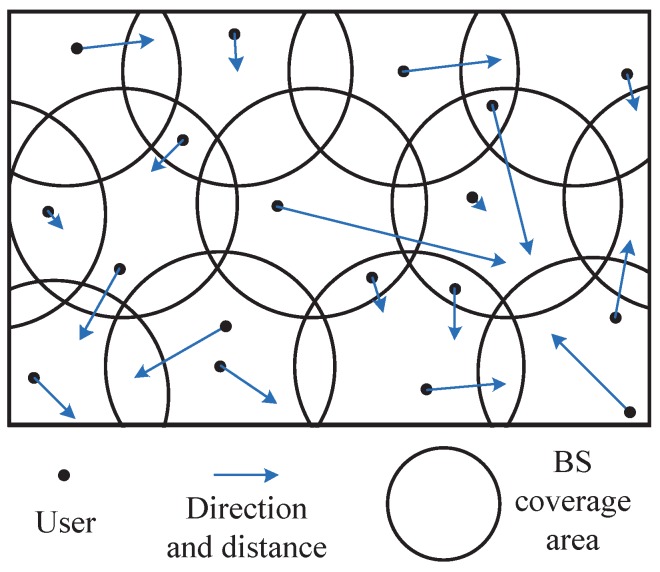
Mobility-Aware Edge Computing Environment.

The traditional edge computing research idealizes the coverage area of each BS as a regular hexagon, while assuming that the user does not move between areas, as shown in Figure 1. However, when we consider user mobility, the overlap of coverage areas must be taken into account. Therefore, we consider the edge computing environment shown in Figure 2, where each circle represents the coverage of the BS, the black dots indicate users, the direction of the user arrow indicates its direction of movement, and the length indicates its speed of movement. First, we make predictions about the user’s target location. When the target location is in the overlapping area, the service request will be forwarded preferentially to the BS which has cached this service. Otherwise, we forward the service request to the edge-cloud server corresponding to the target location as much as possible, thus reducing unnecessary stateful migration [10]. Effective mobile prediction and service allocation can avoid invalid service requests. Then, we propose a service cache selection algorithm based on BP neural network. It can train and predict the most popular services online. The main contributions of this paper are summarized below.

- We propose a new edge-cloud computing model, considering the BS coverage overlap and user mobility, to ensure that user requests are executed as much as possible in local edge server.
- We design a user location prediction model. It uses the existing information to determine the BS where the user is located when the service is completed. Since it is difficult to obtain all the information needed by the model in practical applications, we use frequent patterns to mine local moving track information.
- We propose a service cache selection algorithm based on BP neural network for the edge-cloud server. It uses the historical information and existing service requests to predict the most popular services online.

The rest of the paper is organized as follows. Section 2 deals with related work. We give the system model in Section 3. In Section 4, we describe the user location prediction and service allocation, and Section 5 describes the service cache selection algorithm in edge-cloud based on back-propagation algorithm. The experiment and performance evaluation is show in Section 6. Finally, conclusions and future work are given in Section 7.

## 2. Related Work

Cloud computing platform and gradually developed into the Internet of Things (IoT) application processing efficient platform [11]. However, since the limitations of cloud models and the emergence of highly sensitive IoT applications (e.g., cognitive assistance, image recognition, mobile games and AR/VR), high transmission delays in cloud computing have hampered the development of such IoT applications.

To solve the above problem, the researcher proposed an mobile edge computing (MEC) paradigm [12], the mobile edge device (server) is set at the BS, and the computing job of the mobile device can be offloaded on the edge device. The researchers also proposed many similar concepts, such as cloudlet [13], follow-me cloud [14], fog computing [15], small cell cloud [16] and mobile micro-cloud [17]. Although these different concepts produce slightly different implementations, they all recommend placing a small cloud infrastructure at the edge of the network so that users can seamlessly connect to cloud services. In particular, MECs are typically placed only on one or several network hops from mobile users, thus effectively reducing communication delays [12].

Based on MEC model, researchers have done much research work. The existing research mainly focuses on computation offloading [18,19], Yang et al. [20] proposed to use the user’s mobile mode and service access mode to predict the distribution of user requests in order to minimize the problem of user access delay and service provider cost; and then adjust the service layout and load schedule on the network according to the prediction result. Tan et al. [21] propose a generic model for minimizing job response time in the edge-cloud, where jobs are generated in any order and time on the mobile device. And the first online job scheduling algorithm in the edge-cloud is proposed, called OnDisc algorithm. Wang et al. [17] studied how to offload computing job to deal with the dynamic changes of these networks in the dynamic micro-cloud. The goal is to find the best layout for the instance to minimize the average cost. The paper proposes an offline algorithm that solves the optimal computation offloading problem in a specific look-ahead time window. Wu et al. [22] developed an analytical queueing model for delayed offloading systems with intermittent connectivity. This method effectively improves the success rate of computing offload in a mobile environment. Wu et al. [23] also proposed an energy-efficient offloading-decision algorithm based on Lyapunov optimization. This algorithm effectively reduces the average energy consumption on mobile devices. Although those solutions for computation offloading can effectively solve the problem that mobile device has limited performance. However, the above research does not give a reasonable solution to the problem that what kind of service (database/lib library) should be cached by the edge-cloud to satisfy the near-end service request. Xu et al. [3] studied the dynamic service caching problem in dense cellular networks with MEC enabled. He proposed an OREO algorithm that can jointly optimize dynamic service caching and computational offloading to address many key challenges in MEC systems, including service heterogeneity, unknown system dynamics, spatial demand coupling and decentralized coordination. Chen et al. [9] studied collaborative service caching in MEC-enabled dense small cell (SC) networks. He proposed a collaborative service caching algorithm to solve the service caching problem in a centralized way. However, these existing service cache studies do not consider user mobility. Since users are mobile, the connected edge-cloud will change, and stateful migration will reduce the system performance. Therefore, we need to study a mobility-aware service caching mechanism.

## 3. System Model

We consider the system model that consists of a series of mobile users or mobile sensors, BSs with overlapping coverage (each BS deploys edge-cloud servers) and a remote cloud center. User mobility and BS deployment are shown in Figure 2. To support seamless service transport between MECs, we use the three-tier container architecture described in [10]. The edge-cloud server will co-exist and work with our existing centralized remote cloud. Therefore, we only focus on user mobility prediction and service caching decision, but not on service request forwarding, information interaction and service execution. To simplify the model, we believe that each user can seamlessly connect to the required BS in the process of move.

**Figure 3:**
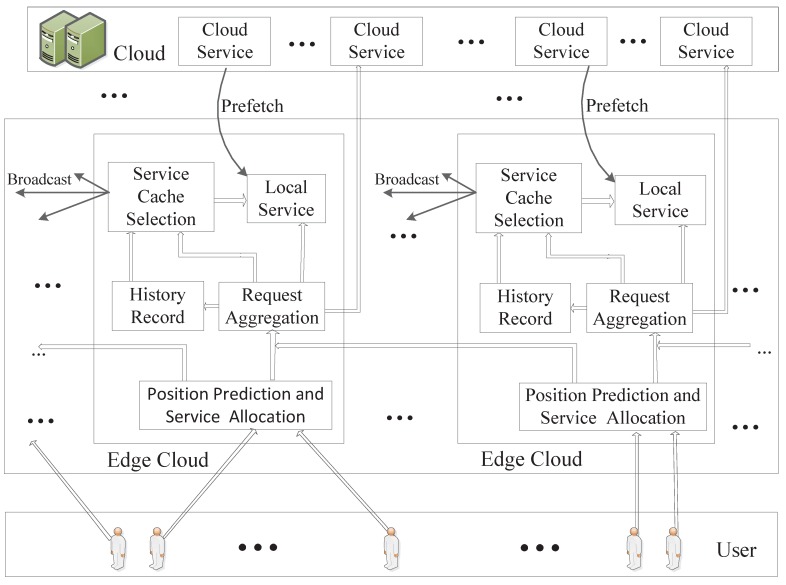
The System Architecture.

The system architecture is shown in Figure 3. Users who can move arbitrarily generate service requests with random probability. The request is first sent to the connected BS, and the edge-cloud server at the location performs mobility prediction. If the predicted user will remain in the range of the BS, subsequent computing jobs are performed locally; otherwise, we will forward the service request to the edge-cloud server corresponding to the target BS according to the service allocation algorithm. In particular, when the target area is completely in the overlapping area, whether to forward the service request is determined by the current service cache of the surrounding BS. After receiving the service request, the edge-cloud server chooses to perform the service locally or forward it to the remote cloud computing center according to whether the service is currently cached. At the same time, the system records and counts the number of all kinds service requests received locally, and predicts the number of service requests in the future based on the established BP neural network model. The neighboring server is notified by broadcast when the edge-cloud server determines the service cache. Our goal is to enable edge-cloud to serve more users.

The entire mobile network contains *U* users, indexed by *U*, each user u∈U; *N* BSs, indexed by *N*, each BS n∈N; and a remote cloud computing center. The system contains *K* services, indexed by *K*, each service k∈K. If the user *u* sends a service request to the edge-cloud deployed by BS *n*, we denote s(u,n,k)=1, otherwise s(u,n,k)=0. Obviously, at any time, the user requests require satisfaction of Equation (1).

(1) $$\sum_{n=1}^{N}\sum_{k=1}^{K}s\left(u,n,k\right)\leq U$$

The computing power of a remote cloud computing center can be seen as infinite and can perform a variety of computing services. The resource required for the cache computing service *k* is $c_{k}$, and the capability of each BS to provide a cache computing service is denoted as $C_{n}$.

(2) $$x_{kn}=\left\{\begin{array}{cc} 0, & \mathrm{computing}\;\mathrm{service}\;k\;\mathrm{is}\;\mathrm{not}\;\mathrm{cached}\;\mathrm{at}\;n\\ 1, & \mathrm{computing}\;\mathrm{service}\;k\;\mathrm{is}\;\mathrm{cached}\;\mathrm{at}\;n \end{array}\right.$$

Obviously, for computing services that can be cached at BS *n*:

(3) $$\sum_{k=1}^{K}x_{kn}c_{k}\leq C_{n}$$

We use $u_{n}^{k}$ indicates the service response time that can be provided to the user *u* when the service *k* is cached by the BS *n*, and $u_{0}^{k}$ indicates the service response time that can be provided to the user *u* when the service is provided by the remote cloud. The service response time is defined as the time from the request generation to the result return.

(4) $$u_{n}^{k}\leq u_{0}^{k}$$

Since the local edge-cloud service response time is less than remote cloud significantly, our goal is maximizing the number of users which served by the local edge-cloud. Our goal is to find the most suitable $\left[x_{kn}\right]$, the objective function of our service caching can be formally expressed as:

(5) $$\begin{array}{cc} \; & \max_{k\in K,n\in N}\;\sum_{u\in U}s\left(u,n,k\right)x_{kn}\\ \; & \;\mathrm{subject}\;\mathrm{to}\;(1)–(4). \end{array}$$

## 4. User Location Prediction and Service Allocation

For the user mobility problem, first, we propose an idealized geometric model to predict the target area of the user’s movement. Then, considering that it is difficult to obtain all the data needed for the model in practical applications, we use frequent patterns to mine local moving track information. Finally, we propose a service allocation algorithm that forwards service requests to the target area as much as possible while considering the existing service cache.

### 4.1. Idealized Geometric Model

To predict the target area of the user’s movement, we need to obtain the user’s current location information and motion status. We represent the obtained information as the following 6-tuple:(Position, Position error, Speed, Speed error, Direction, Direction error). Among them, the Position is obtained by the positioning system, the Position error is generated according to the influence of the positioning system and the environment; the Speed refers to the average speed from the current position to the service completion position, and the Speed error is determined by the motion mode and the speed accuracy of the positioning system; similarly, the Direction indicates the angle from the current position to the position at which the service is completed. Since the service we are considering is completed in a relatively short time, we believe that the Direction is a linear direction and the Direction error is the possible direction deviation.

It should be noted that this paper focuses on the service cache using the mobility prediction results. We do not consider how to obtain user mobility information, the protection of personal location privacy and how to complete information estimation when relevant information is not available. Relevant researchers have provided mature solutions to the above problems, and interested researchers can refer to [24,25,26,27,28].

**Figure 4:**
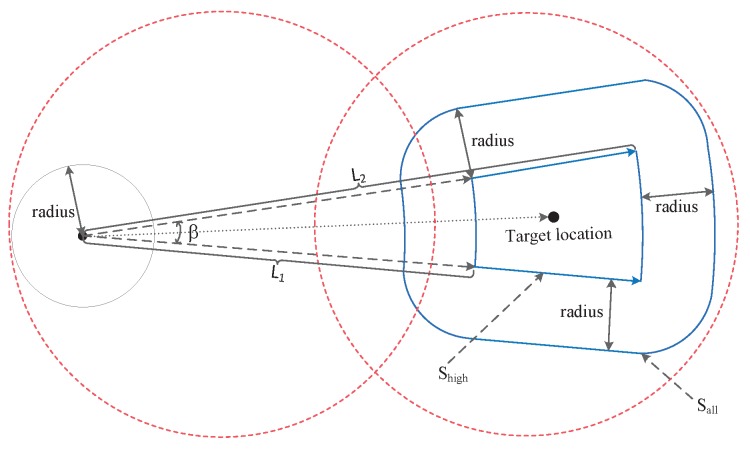
User Location and Target area.

As shown in Figure 4, the blue area is the possible position after the user moves, radius is the size of the position precision, represented as rad; $L_{1}$ represents the shortest moving distance (estimated service response time * minimum moving speed), and $L_{2}$ represents the maximum moving distance (estimated service response time * maximum moving speed). β is the angle of direction error twice. The position accuracy and speed accuracy provided by the existing positioning system (including the one under construction) are given in Table 1. In this paper, we use GPS performance parameters, and all the above performance parameters are civil performance parameters [29].

Therefore, the area of the target area that the user may move to can be approximated as follows:

(6) $$S_{all}\approx \pi *rad^{2}+\frac{\beta *\pi }{360}\left[{\left(L_{2}+rad\right)}^{2}-{\left(L_{1}-rad\right)}^{2}\right]+2*rad*\left(L_{2}-L_{1}\right)$$

Among them, the middle small fan shape is a region with a higher probability of the user’s location, and the area can be expressed as:

(7) $$S_{high}=\frac{\beta *\pi }{360}\left(L_{2}^{2}-L_{1}^{2}\right)$$

The process by which we calculate the target location and target area is as follows.

**step1:** Calculate the target location using position, direction, speed, and estimated service response time.

**step2:** Calculate $S_{all}$ by Equation (6).

**step3:** Calculate $S_{high}$ by Equation (7).

**step4:** Find all the BSs near the target area through the target location and $S_{all}$, denoted as $B=\{n_{1},n_{2},\ldots ,n_{all}\}$.

### 4.2. Mining Frequent Patterns

To use the geometric model proposed in the previous section to predict the target location, we need to collect the user’s movement track information, i.e., the 6-tuple information. However, in practical applications, it is often difficult to obtain all the information due to the unreliability of the mobile device and the limitations of the positioning system. Therefore, we use the data mining frequent pattern to analyze the trajectory data in the same range to obtain the common behavior pattern at the group level. In this paper, we study the potential association rules of user movement trajectory information, so that the trajectory information can be complemented for the model to perform location prediction.

Our basic assumption is that people often follow the crowd: individuals tend to follow a common path. For example, people go to work every day through similar routes, pedestrians walk at similar speeds and public transportation goes through similar routes in different time periods [30]. Therefore, if we have enough data to simulate typical behavior, we can use this knowledge to predict the missing user movement information for most people. Existing research has proved that this assumption is reasonable [30]. In this paper, we use the movement trajectory of all users/objects in the base station area to learn motion information.

**Figure 5:**
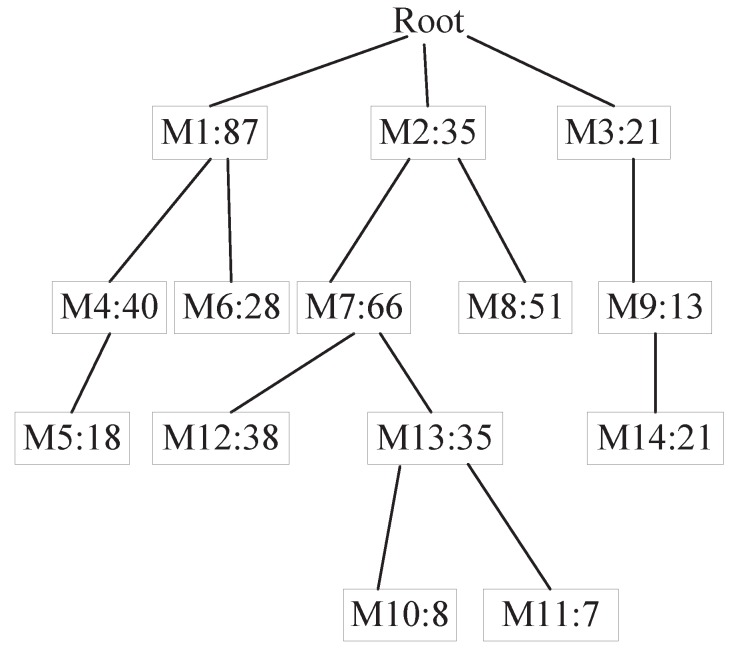
T-pattern Tree.

For position predictions where all mobile information has been acquired, we directly use the geometric model presented in the previous section. If the information obtained is incomplete, we build T-pattern Tree [31]. The T-pattern Tree constructed is shown in Figure 5. The Root is a virtual node. And the number in the node indicates the number of supports. We use the existing mobile information as a transaction data set. The element items in the transaction include the movement track information. First, we traverse the data set, calculate the frequency of each individual element, and filter out the unsatisfied elements based on the minimum support. Then, the data set is processed. Each piece of data is sorted by the absolute frequency of occurrence of the elements and filters out elements that do not satisfy the minimum support. After processing, iterate through the data set, insert each piece of data into the T-pattern Tree, and recursively add the path from the root node. If it exists, the value will be increased. If it does not exist, a new node will be created. We can find frequent item sets by looking up the condition base of the element item. Repeat the above process until the T-pattern Tree contains only one element [32]. This element is substituted into the model as a missing element to predict the user’s location.

### 4.3. Service Allocation Algorithm

**Figure 6:**
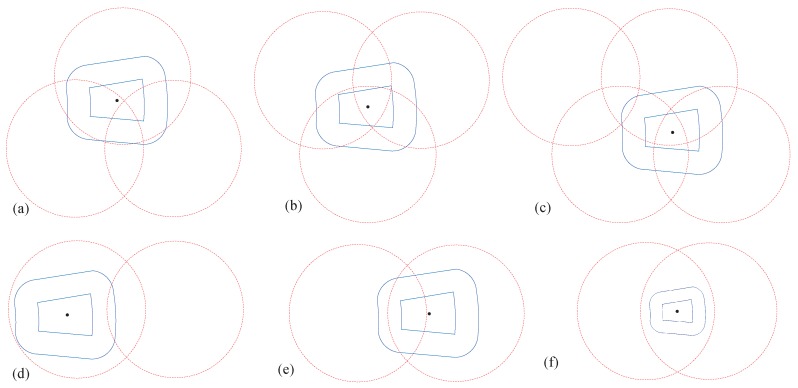
Target area and BS coverage area.

Due to the uncertainty of the location and direction of movement when the user requests the service, the area where the user may eventually appear has multiple cases. Figure 6 shows some of the possible cases. How to forward the service requests in different cases is a problem that we need to solve.

To send the service request to the most suitable edge-cloud server, we design a service allocation algorithm based on existing service cache. When the user is in the overlapping area, the service request is preferentially forwarded to the BS which has cached this service. Otherwise, by calculating the proportion of target area within the coverage of each base station, the BS with high probability is selected to provide services in priority. The service allocation algorithm is shown in Algorithm 1. Among them, Max represents the maximum iteration number.
**Algorithm 1** Service Allocation Algorithm1:Initialize Max = 0.2:**if**$S_{high}$ is all in the overlap area **then**3: **for** $n_{i}$ = $n_{1}$ to $n_{all}$ **do**4:  **if** $n_{i}$ has cached this service **then**5:   target BS = $n_{i}$;6:   Send request to $n_{i}$;7:   break;8:  **end if**9: **end for**10:**else**11: **for** $n_{i}$ = $n_{1}$ to $n_{b}$ **do**12:  $A(n_{i})$ = the size of $S_{all}$ in $n_{i}$13:  **if** $Max<A(n_{i})$ **then**14:   $Max=A(n_{i})$;15:   target BS = $n_{i}$;16:  **end if**17: **end for**18: Send request to target BS.19:**end if**

We give priority to the distribution of the $S_{high}$ area. When $S_{high}$ is all in the overlap area, we prefer to forward the request to the nearby base station which has cached this service. We denote the size of $S_{all}$ in base station $n_{i}$ as $A(n_{i})$. When $S_{high}$ is not all in the overlap area, we select the BS which has largest $A(n_{i})$ as the target BS.

## 5. Service Cache Selection Algorithm

Edge-cloud servers can aggregate to more accurate local service requests through mobility prediction. And it also avoids invalid service requests. Using the aggregated service requests to accurately predict the amount of service requests is the key to determine the service cache. Since the trends of service requests from different locations are different, we need to make predictions on service requests online. Considering that the edge server has limited computing resources, we must use a simple and efficient model for prediction.

BP neural network effectively meets the above requirements. In this paper, first, we use historical data to count the top *k* service types of local edge server service requests. After that, the BP neural network model is used to predict the number of the above *k* service requests in the future. Finally, the service cache is implemented based on the predicted results.

### 5.1. BP Neural Network Model

The BP network implements the mapping function from input to output, which can prove that the three-layer neural network can approximate any nonlinear continuous function with arbitrary precision, which makes it especially suitable for solving complex internal mechanisms [33]. At the same time, since it can automatically extract the “reasonable rules” between input and output data through learning, and adaptively store the learning content in the weight of the network. Therefore, we can use it to predict the number of requests for a certain service type in the future [34].

In contrast to data caching, service caching usually needs to consider the long-term behavior characteristics of the server. To ensure the stability of the edge server, we cannot change the cached service frequently. Therefore, we start each hour with a prediction of the service for the next hour and cache the service based on the prediction. We use 24 h as a cycle, denoted by *h*. According to [35], we can obtain prior knowledge that the request frequency of different services is related to whether it is a working day. Therefore, we consider the pattern of service requests at different periods in a week, using d=1,2…,7 to represent Monday to Sunday.

**Figure 7:**
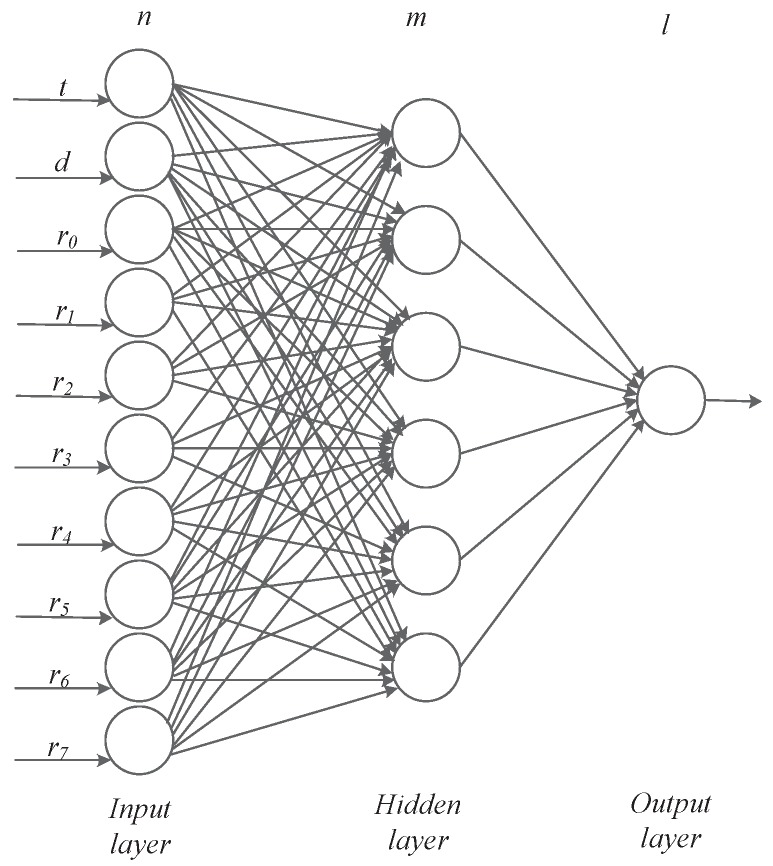
BP Neural Network Model.

As shown in Figure 7, the BP network that we constructed consists of an input layer, a hidden layer and an output layer. It is a 10×m×1 three-layer BP network model. When constructing a BP neural network model, it is important to determine the number *m* of hidden neurons. Excessive number of hidden layer neurons will increase the amount of network calculation and easily cause over-fitting problems; if the number of neurons is too small, it will affect network performance and will not achieve the expected results. The number of hidden layer neurons in the network is directly related to the complexity of the actual problem, the number of neurons in the input and output layers, and the setting of the expected error. In this paper, we use the following empirical formula to select the number of hidden layer neurons:

(8) $$m=\sqrt{n+l}+\lambda$$

where λ is a constant between 1–10, *n* is the number of input layer nodes, and *l* is the number of output layer nodes.

Therefore, we choose m=6 as the number of nodes in the hidden layer. We have 10 neurons in the input layer and one neuron in the output layer, and the output is the number of service requests in the next period. $W^{l}$ represents a matrix, *b* is the offset vector, and *L* is the *L*-th layer. $A^{l}$ represents the output value of the *l*-th layer. We use the mean square deviation to construct the loss function, hoping to minimize Equation (9).

(9) $$J\left(W,b,In,Out\right)=\frac{1}{2}{∥a^{L}-Out∥}_{2}^{2}$$

where In represents the input 10-dimensional vector, $a^{L}$ represents the calculation result of the *L*-th layer of the output layer, and Out represents the desired output result. ∥S∥ indicates the L2 norm of *S*. The network model uses the Sigmoid activation function, as shown in Equation (10):

(10) $$f\left(x\right)=\frac{1}{1+e^{-x}}$$

We use the gradient descent method to iteratively solve *W*, *b* for each layer. Since $a^{L}=\sigma \left(z^{L}\right)=\sigma \left(W^{L}a^{L-1}+b^{L}\right)$, we can get:

(11) $$\frac{\partial J(W,b,In,Out)}{\partial W^{L}}=\frac{\partial J(W,b,In,Out)}{\partial Z^{L}}\frac{\partial Z^{L}}{\partial W^{L}}=\left(a^{L}-Out\right)*\sigma^{\prime }\left(Z^{L}\right){\left(a^{L-1}\right)}^{T}$$

(12) $$\frac{\partial J(W,b,In,Out)}{\partial b^{L}}=\frac{\partial J(W,b,In,Out)}{\partial Z^{L}}\frac{\partial Z^{L}}{\partial b^{L}}=\left(a^{L}-Out\right)*\sigma^{\prime }\left(Z^{L}\right)$$

(13) $$\delta^{L}=\frac{\partial J(W,b,In,Out)}{\partial Z^{L}}=\left(a^{L}-Out\right)*\sigma^{\prime }\left(Z^{L}\right)$$

After that, we can recurse the gradient of the front layer. We use the following formula in forward propagation:

(14) $$z^{l}=W^{l}a^{l-1}+b^{l}$$

Therefore, the gradients of $W^{L}$ and $b^{L}$ of the *L*-th layer are as follows:

(15) $$\frac{\partial J(W,b,In,Out)}{\partial W^{L}}=\frac{\partial J(W,b,In,Out)}{\partial Z^{L}}\frac{\partial Z^{L}}{\partial W^{L}}=\delta^{l}{\left(a^{l-1}\right)}^{T}$$

(16) $$\frac{\partial J(W,b,In,Out)}{\partial b^{L}}=\frac{\partial J(W,b,In,Out)}{\partial Z^{L}}\frac{\partial Z^{L}}{\partial b^{L}}=\delta^{l}$$

Obviously, the recursive relation of $\delta^{l}$ can be obtained by inductive method:

(17) $$\delta^{l}=\delta^{l+1}\frac{\partial z^{l+1}}{\partial z^{l}}={\left(W^{l+1}\right)}^{T}\delta^{l+1}*\sigma^{\prime }\left(z^{l}\right)$$

The training process of the predictive model based on the back-propagation algorithm is shown in Algorithm 2. We use the batch gradient descent method. The input 10-dimensional vectors represent clock *h*, day of the week *d*, last time period request quantity $r_{0}$, historical synchronous request quantity $r_{1},r_{2},r_{3},r_{4},r_{5},r_{6},r_{7}$.
**Algorithm 2** BP Neural Network Training Model1:Initialize coefficient matrix W and the offset vector b as a random value;2:**for** iteration number = 1 to MAX **do**3: **for** i=1 to *Y* **do**4:  Set input $a^{1}$ as $In_{i}$5:  **for** l=2,3 **do**6:   Calculation $a^{i,l}=\sigma \left(z^{i,l}\right)=\sigma \left(W^{l}a^{\left(l-1\right)}+b^{l}\right)$;7:  **end for**8:  Calculate the output layer’s $\delta^{i,3}$ through the loss function;9:  Calculate $\delta^{i,2}={\left(W^{3}\right)}^{T}\delta^{i,3}*\sigma^{\prime }\left(z^{i,2}\right)$10:  Update $W^{2}$ and $b^{2}$:11:   $\;W^{2}=W^{2}-\alpha \sum_{i=1}^{Y}\delta^{i,2}{\left(a^{i,1}\right)}^{T}$12:   $\;b^{2}=b^{2}-\alpha \sum_{i=1}^{Y}\delta^{i,2}$13:  **if** The change value of W,b≤ϵ **then**14:   break;15:  **end if**16: **end for**17:**end for**

### 5.2. Service Cache Selection Algorithm

Based on the above training results, the coefficient matrix *W* and offset vector *b* can be obtained. Therefore, we can predict the number of future service requests and design the corresponding service caching algorithm.

We use the historical data to count the top *k* service types of local service requests, denoted as top[k]. Then, using Algorithm 2, at the beginning of each time period, predict the requests for the top *k* services and sort the requests. Finally, the first *i* services are cached according to the sorting result, where *i* represents the maximum number of cacheable services that the current base station can provide.

(18) $$\sum_{i=1}^{i}c_{i}\leq C_{n}$$

In this way, the number of users supported by the locally cached service can be maximized. The pseudo code of the service cache selection algorithm which we design is shown in Algorithm 3.
**Algorithm 3** Service Cache Selection Algorithm**Require:**W,b,k, local historical data. 1:Select the top *k* services with the largest number of local service requests, denoted as top[k];2:**while** each time period begins **do**3: Initialize $c_{i}=0$;4: **for** j=0;j<k;j++ **do**5:  Get $Out_{j}$ with trained BP neural network;6: **end for**7: Sort the resulting $Out_{j}$ in descending order, denoted as top[j];8: **for** j=0;j<k;j++ **do**9:  **if** $c_{i}\leq C_{n}$ **then**10:   Cache service top[i];11:   $c_{i}=c_{i}+c_{j}$;12:  **else**13:   break;14:  **end if**15: **end for**16:**end while**

### 5.3. Model Performance Analysis

To choose a suitable prediction model, we have compared the performance of the 3-layer BP network model, the deep neural network (DNN) model, and the recurrent neural network (RNN) model. The model performance comparison is shown in Table 2. Among them, MAE represents the mean absolute error, RMSE represents the root mean squared error.

It can be seen that the MAE of the three models is almost the same. However, the RMSE of DNN and RNN models is higher than the RMSE of 3-layer BP model. In addition, it also can be seen from Table 2, the iteration number of RNN and DNN is significantly higher than the iteration number of 3-layer BP. Therefore, the proposed 3-layer BP model is much more suitable for service cache prediction.

## 6. Experiment and Performance Evaluation

To verify the effectiveness of the proposed algorithm, we take a series of experiments. It is hard to acquire real service datasets due to the users’ data confidentiality issues and policies maintained by service providers (SP) [36]. Therefore, we adopt the Baidu API to obtain the points of interest in each area, and then determine the type of service request in the base station. The service request obeys Poisson distribution. We used the GPS trajectory dataset from Microsoft Research Asia [37,38,39] and user trajectories extracted from Call Detail Records (CDR) which published by Orange [40] and the University of Minnesota [41] to construct the dataset required for the experiment.

### 6.1. Experimental Environment

We conduct systematic simulations in practical scenarios, and our system model adopts the widely used stochastic geometry approach. We choose a 15 km × 7 km rectangular area in the north of Beijing as the simulation experiment area. We assume that the BS coverage radius is 500 m and the distance between adjacent BSs is 750 m. The user’s starting position is randomly generated, and the user’s moving distance and time are generated from previously constructed dataset.

**Figure 8:**
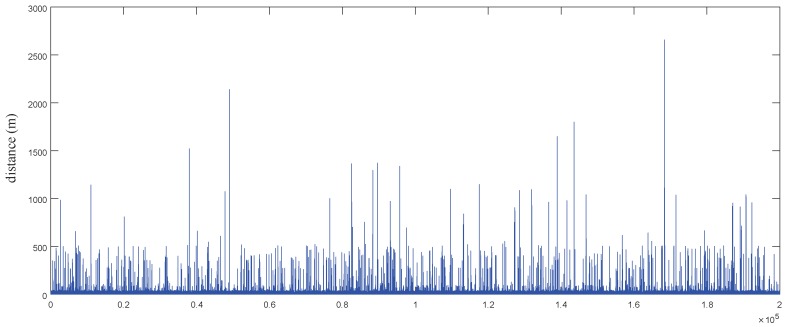
Moving Distance Distribution.

The dataset is represented by a sequence of time-stamped points, each of which contains the information of latitude, longitude, high, speed and heading direction, etc. To construct the experimental data set, we use the spherical body distance formula to calculate the distance between adjacent points. Here, we take 6,371,000 m as the radius of the earth. We refer to the trajectory of adjacent points in the data set as the moving path, and randomly select 200,000 moving paths. The moving distance distribution is shown in Figure 8. The y-axis represents the moving distance and the x-axis represents the track number. It can be seen that the length of most trajectories does not exceed 1 km.

**Figure 9:**
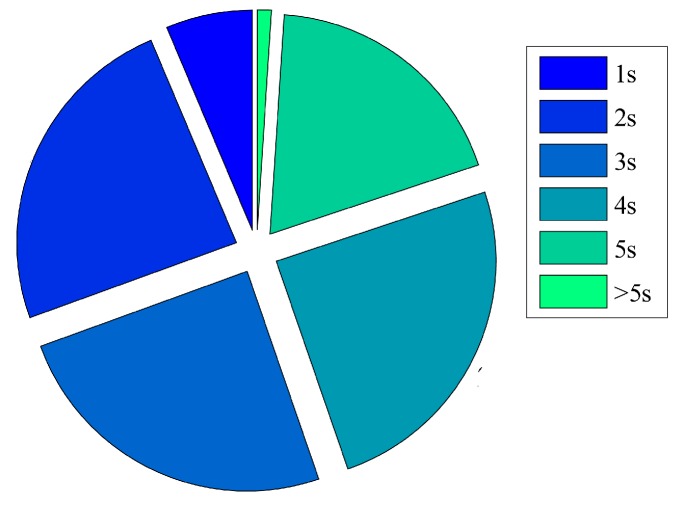
Service Execution Time.

To facilitate the calculation, we use the user’s movement time between two points as the service execution time. The distribution of service execution time is shown in Figure 9. Among them, 81.88% of the movement time is less than 5 s.

As described in [42], the main traffic type under a BS is strongly correlated with the location of the BS and points of interest (POIs) around. Inspired by this, we use POIs around BSs to determine the main type of service request within the BS. Service type is Zipf-like distribution. The frequency *F* of any service is inversely proportional to its rank in the frequency table *r*. It can be expressed as $F=C/r^{z_{p}}$, where *C* denotes the normalization factor, $z_{p}$ is a parameter. Since the different $z_{p}$ values imply the different content distribution, we take experiment to evaluate the algorithm performance under different $z_{p}$ values [43]. In our experiments, $z_{p}$ is set to 0.5, 0.7, and 0.9, respectively.

The delay from terminal device to the edge server is set between 0.02 s and 0.05 s, and the delay from the terminal device to the remote cloud server is set between 0.4 s and 1 s. We assume that all the edge-cloud serves are the same, and there are a total of 100 different types of services. If not specified explicitly, we assume that the cache space in the edge-cloud server can cache 10 different services.

### 6.2. Performance Comparison

In the comparison experiment, we used three different algorithms to compare with our method (MASC). **Service caching strategy without mobility-aware (Non-MASC):** BSs cache services are determined by our service cache selection algorithm, but lack user mobility prediction. The service request is completed by the edge-cloud server at the starting location. **Myopic service caching (MSC) [3]:** This method has user mobility prediction. However, it does not consider long-term service request rules, and the cache services is only determined by the request amount of the previous period. **Collaborative service caching (CSC) [9]:** Collaborative service caching assume that SCs are obedient and fully cooperative. The service caching decisions of the SCs will reshape the workload distribution in the network to better use the limited computing resources of individual BSs. If the service is not executed locally, it needs to generate multi-hop data transmission. In this case, we assume that the data transmission delay between different base stations is 0.5 times the execution time.

We divide all the service request into separate data groups. In each experiment, we randomly select 20,000 moving tracks from 200,000 paths. We evaluate the performance of the service caching algorithm from the average service response time and local service proportion. Among them, the local service proportion refers to the proportion of service requests completed by the local edge-cloud server.

**Figure 10:**
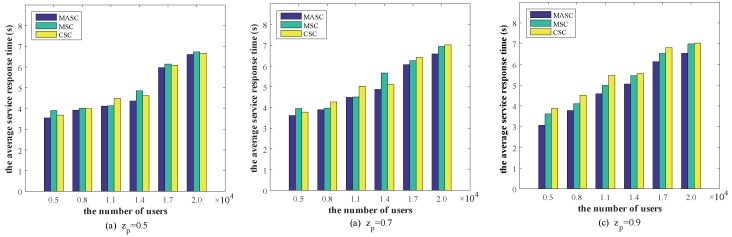
Performance comparison under different user numbers.

To verify the performance of our service cache selection algorithm, we compare MASC, MSC, and CSC algorithms. Figure 10 depicts the performance comparison of different algorithms under different service request distributions, where the x-axis denotes the number of users and the y-axis denotes the average service response time.

We observe that when the number of users increased from 5000 to 20,000, the average service response time has increased. Especially when the number of users exceeds 14,000, the average service response time increases significantly. This shows that after the number of users exceeding the edge server capacity, many service requests are forwarded to the remote cloud for execution. Moreover, it can be seen in Figure 10c, the average service response time when $z_{p}=0.9$ is significantly less than the other two cases. This means that our algorithm performs better when the service request obeys long-tailed distributions.

By comparing the experimental results when $z_{p}=0.9$, the proposed Mobility-Aware Service Caching Strategy can save the service response time by about 13.21% on average compared to the collaborative service caching. Compared to the Myopic Service Caching algorithm, it can save the service response time by about 8.46% on average, the biggest gap is 14.95%. Therefore, our algorithm is significantly better than other algorithms.

**Figure 11:**
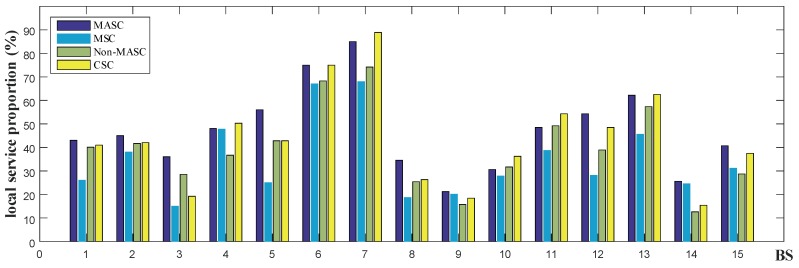
Performance comparison ($z_{p}=0.8$).

To verify the performance of our mobility prediction and service allocation algorithms, we compare MASC, Non-MASC, MSC, and CSC algorithms. Figure 11 depicts the performance comparison under different BSs, where the x-axis denotes the BS and the y-axis denotes the local service proportion. We selected 15 BSs with more service requests for comparison. In this experiment, $z_{p}$ is set to 0.8 and the number of users is set to 12,000.

As shown in Figure 11, the MASC algorithm is significantly better than the MSC algorithm, with an average 45% higher than the MSC algorithm. Compared with the CSC algorithm, the local service proportion of the MASC algorithm is higher in most locations. Only in a few locations, such as BS 4,7,10,11, the local service proportion of our algorithm is slightly lower than the CSC algorithm. This is caused by the service load being forwarded to nearby cooperating edge servers. However, this significantly increases service response time (as shown in Figure 10). And the local service proportion of our proposed MASC algorithm is 15.19% higher on average than the CSC algorithm. Therefore, our algorithm is still better than the CSC algorithm.

We observe that our algorithm performs significantly better than the Non-MASC algorithm in most cases. And we also find that in a few locations, such as BS 10 and BS 11, the performance of our proposed algorithm is almost the same as the Non-MASC algorithm. Through analysis, we find that it is due to the complex road network within the range of the BS, which is difficult to effectively complete the prediction of the moving trajectory. However, the local service proportion of our proposed MASC algorithm is 23.6% higher on average than the Non-MASC algorithm, the biggest gap is 30.8%. Therefore, the performance of our algorithm is higher than the Non-MASC algorithm.

To observe the impact of mobility prediction on service cache performance, we select different trajectory data for comparison experiments. First, we filter the trajectory with the moving distance greater than 250 m from the Microsoft Research Asia’s GPS Trajectories dataset. Then, we add non-mobile users to the dataset in proportions of 10%, 20%, 30%, 40%, 50%, 60%, and 70%, respectively. Non-mobile users mean that the user is in a fixed location, it receives and generates information that is always in the same location. Finally, we compare the performance of MASC and Non-MASC algorithms under different non-mobile user proportions. In this experiment, $z_{p}$ is set to 0.8.

**Figure 12:**
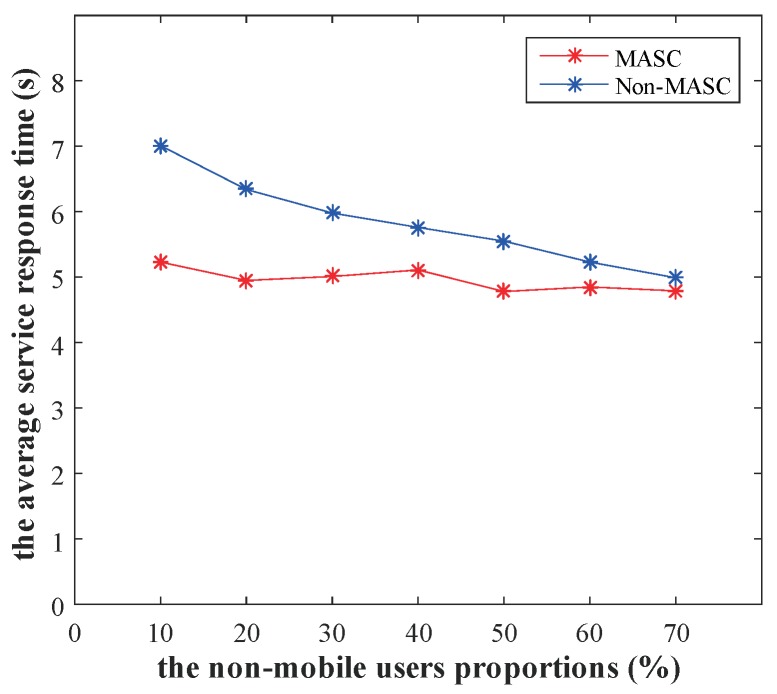
Performance comparison under different non-mobile user proportions ($z_{p}=0.8$).

Figure 12 depicts the performance comparison under different non-mobile user proportions, where the x-axis denotes the non-mobile user proportions and the y-axis denotes the average service response time.

As shown in Figure 12, the average service response time of the MASC algorithm is stable at different proportions. And the service response time of Non-MASC algorithm is significantly larger than the MASC algorithm when the non-mobile user proportion is less than 70%. The Non-MASC algorithm lacks mobility prediction and service allocation, resulting in many service requests being executed in non-local areas or retransmitted. Therefore, our algorithm has good performance for environments with high user mobility.

## 7. Conclusions and Future Work

In this paper, we focus on the service caching strategy in IoT. The strategy takes user mobility into account. Based on the user location and mobility information, the base station forwards the service request to the target area as much as possible. In addition, we propose a service allocation algorithm to ensure that service is completed by the appropriate edge-cloud server, when the user is in the overlapping areas. Finally, we propose a service cache selection algorithm based on BP neural network, and choose the appropriate service for caching.

By comparing the experimental results, the proposed Mobility-Aware Service Caching Strategy can save the service response time by about 13.21% on average compared to the collaborative service caching. Compared to the Myopic Service Caching algorithm, it can save the service response time by about 8.46% on average. The local service proportion of our proposed MASC algorithm is 15.19% higher on average than the CSC algorithm and an average 45% higher than the MSC algorithm. The simulation results show that our method can effectively reduce the service response time and increase local service proportion. On the one hand, service allocation algorithm and user location prediction effectively avoid the situation of request failure. On the other hand, effective service predictions are made for the service cache, so the edge server can serve more users for a considerable period of time.

In the future, we will optimize the user location prediction, especially in the absence of GPS location information. Moreover, we will further look for a suitable service request dataset to train the neural network model with real data. In addition, we will explore the prediction algorithm for service cache sequence.

## Figures and Tables

**Table 1:** Positioning system performance comparison.

Name	Position Accuracy	Timing Accuracy	Speed Accuracy
GPS	2–10 m	20 ns	0.1 m/s
Galileo	1–5 m	20 ns	0.1 m/s
Glonas	10–20 m	25 ns	0.1 m/s
Beidou	5–15 m	50 ns	0.2 m/s

**Table 2:** Model Performance Comparison.

Kind	Group	Iterations	MAE	RMSE
3-layer BP
	1	2000	26.51	29.17
	2	895	15.17	17.56
	3	1118	26.82	29.25
	4	1690	7.53	8.86
	5	2000	13.86	15.43
	average	1540.6	18.178	20.054
DNN
	1	2000	18.92	24.35
	2	1083	26.57	34.58
	3	1601	32.1	43.57
	4	2000	8.98	11.13
	5	1920	6.52	8.52
	average	1720.8	18.618	24.43
RNN
	1	1575	13.74	19.04
	2	1119	18.98	25.36
	3	2000	22.45	29.81
	4	2000	14.83	18.18
	5	2000	21.17	23.56
	average	1738.4	18.234	23.19
